# The impact of mind–body exercise on the quality of life in older adults: the chain mediation effect of perceived social support and psychological resilience

**DOI:** 10.3389/fpubh.2024.1446295

**Published:** 2024-10-03

**Authors:** Qingqing Yang, Yinkai Zhang, Shiying Li

**Affiliations:** ^1^Chinese Wushu Academy, Beijing Sports University, Beijing, China; ^2^School of Humanities, Beijing Sport University, Beijing, China

**Keywords:** mind–body exercise, perceived social support, psychological resilience, quality of life in older adults, the chain mediation effect

## Abstract

**Background:**

With the intensification of the global aging trend, there is a contradiction between the extended lifespan and the decline of physiological functions among the older adult. It has become a global consensus to focus on and improve the quality of life for the older adult. Mind–body exercises (Tai Chi, Ba Duan Jin, Yi Jin Jing) play a crucial role in promoting the quality of life for older adults, but the mechanisms and mediating effects are not yet clear.

**Objective:**

This study examines the impact of mind–body exercises (Tai Chi, Ba Duan Jin, Yi Jin Jing) on the quality of life in older adults, with a particular focus on exploring the chain mediating effects of perceived social support and psychological resilience.

**Methods:**

This study is a cross-sectional study that surveyed 1,087 older adults participating in mind–body exercises (Tai Chi, Ba Duan Jin, Yi Jin Jing) in 13 districts of Beijing, China, from March 25 to May 3, 2024. The Physical Activity Rating Scale (PARS-3), the World Health Organization Quality of Life Scale (WHOQOL-BREF), the Perceived Social Support Scale (PSSS), and the Connor-Davidson Resilience Scale (CD-RISC) were used to measure mind–body exercise, perceived social support, psychological resilience, and quality of life, respectively. Data were statistically analyzed using SPSS 26.0, and mediation effects were tested and effect analysis was conducted through structural equation modeling (AMOS) and the Bootstrap method.

**Results:**

The study results show that mind–body exercises (Tai Chi, Ba Duan Jin, Yi Jin Jing) are significantly and positively correlated with the quality of life in older adults (*r* = 0.549, *p* < 0.01). The path coefficients for the relationships mind–body exercise → perceived social support (*β* = 0.46, *p* < 0.001) → psychological resilience (*β* = 0.20, *p* < 0.001) → quality of life in older adults (*β* = 0.39, *p* < 0.001) are significant, indicating that perceived social support and psychological resilience have a chain mediating effect between mind–body exercise and the quality of life in older adults.

**Conclusion:**

Mind–body exercises not only improve the quality of life for older adults but also indirectly enhance it by strengthening perceived social support and psychological resilience. This study provides significant reference for developing health intervention strategies targeted at older adults, suggesting that promoting mind–body exercises can improve their sense of perceived social support and psychological resilience, thereby increasing their quality of life.

## 1 Introduction

The issue of global aging is becoming increasingly severe, with old age being a period of decline in the life cycle of an individual. Older adults must face various stressors that are distinct from those experienced by other age groups, such as limitations in daily activities (1), an increase in chronic diseases (2), changes in social roles (3), and a decrease in self-worth (4). These stressors can all potentially threaten the Quality of Life in older adults (5, 6). A decline in the Quality of Life in older adults can subsequently increase family stress and socio-economic burdens, leading to a variety of social issues. Ensuring the Quality of Life in older adults has become a focal point of concern for the public and the health system.

Existing literature indicates that physical exercise is the most effective, green, and scientific method to enhance the quality of life for older adults, and it can have a positive long-term impact on both physical function and psychological state (7, 8). Mind–body exercise, as a form of physical activity that integrates mental concentration, breath control, and physical movement, possesses characteristics of both aerobic and resistance exercises, making it particularly suitable for older adults (9). Regular and rigorous engagement in mind–body exercise is essential. Numerous studies have shown that appropriate mind–body exercise, especially proactive physical activity, can delay degenerative changes and improve the quality of life and health (10, 11). Furthermore, mind–body exercise also significantly enhances individual psychological health. By strengthening emotional regulation capabilities, it can effectively alleviate psychological stress, depression, and loneliness caused by factors such as family, society, and retirement (12), thereby maintaining a good mental state and enhancing their evaluation of overall quality of life.

The influence of mind–body exercises on the quality of life in older adults is not confined to a single dimension but is subject to the mediating effects of multiple factors, thus constituting a multi-tiered and dynamic mechanism of action. Quality of life encompasses multiple dimensions, including material, psychological, and social aspects, among which the state of mental health is a significant component of the overall quality of life (13). A positive mental health status is crucial for the older adult to enjoy a happy later life and promote harmonious social development. Perceived social support is an essential psychological resource for the older adult, which is closely associated with the quality of life (14). Psychological resilience is one of the components of the mental health quality system (15), and high psychological resilience can help the older adult improve their quality of life, even when they may face adversity (16). Previous research results indicate that the level of perceived social support among older adults has an impact on their level of psychological resilience (17). Therefore, to achieve a more comprehensive analysis, this study incorporates perceived social support and psychological resilience, and by constructing a chained mediation model, it thoroughly investigates the complex interrelationships between mind–body exercise, perceived social support, psychological resilience, and the quality of life in older adults, with the aim of offering insights to facilitate healthy aging in this population.

## 2 Statement of the study

In recent years, with the intensification of the aging population issue, how to enhance the quality of life for older adults has become a focal point of societal concern. Mind–body exercises have a significant positive impact on the quality of life in older adults, while psychological factors, such as perceived social support and psychological resilience, also play a crucial role in fostering a positive lifestyle among older adults. Existing literature mostly focuses on whether older adults’ participation in mind–body exercises has a positive impact on their quality of life (18, 19), with more emphasis on the changes in physical functioning that occur after older adults practice mind–body exercises (20, 21). However, there is less exploration of the roles of these mediating variables in the process, especially the chained mediating effects of perceived social support and psychological resilience, which have not been fully validated. Therefore, this study aims to fill this research gap by exploring the indirect impact of mind–body exercise on the quality of life in older adults through perceived social support and psychological resilience. Specifically, this study proposes a chained mediation model, attempting to reveal how mind–body exercise can enhance the perceived social support of older adults, thereby strengthening their psychological resilience and ultimately improving their quality of life.

**Figure 1:**
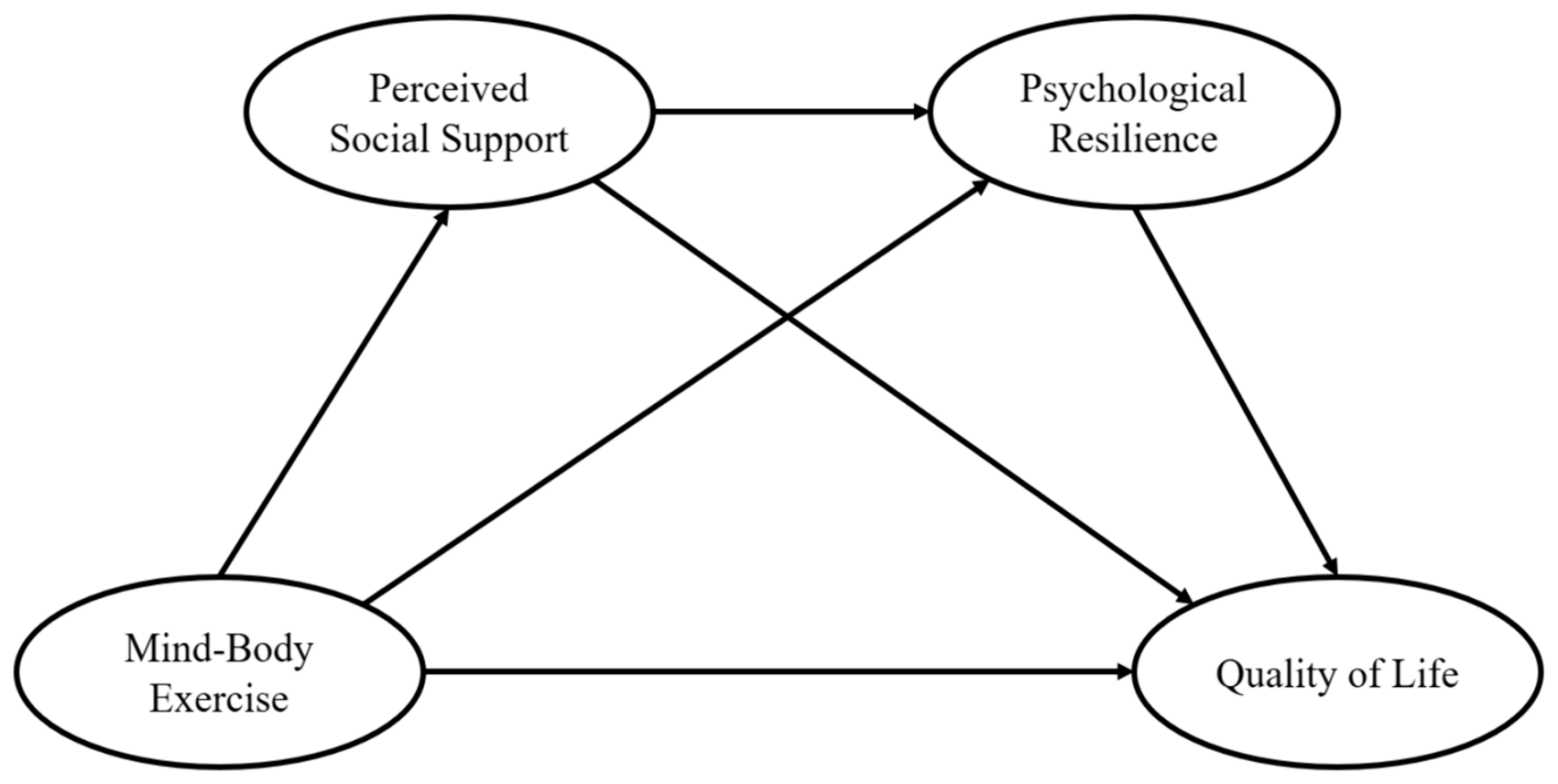
A hypothetical model of mind–body exercise affecting quality of life.

The theoretical framework of this study is based on Rutter’s Developmental Model (RDM), which emphasizes that individuals develop coping abilities through the interaction of external and internal protective factors when facing stress, adversity, or challenges, thereby enhancing adaptive capacity and mental health, and ultimately improving their quality of life (22). Mind–body exercise, as an external protective factor, can not only enhance the physical function of older adults, improve cardiopulmonary function, increase balance ability, and alleviate health issues related to aging and chronic diseases, but it can also directly promote the improvement of the quality of life by regulating emotions and increasing life satisfaction (23). Perceived social support, as another significant external protective factor, refers to an individual’s subjective feelings and evaluations of the degree to which they are supported by external sources, including family support, friend support, and other forms of support (24). Perceived social support can provide emotional backing and practical assistance to older adults, alleviating their feelings of loneliness and stress in life, thereby helping to maintain a higher quality of life. Psychological resilience is a key internal protective factor in the RDM, referring to an individual’s ability to adapt and recover when facing adversity (25). Psychological resilience helps older adults maintain a good psychological state and enhance life satisfaction by actively adapting to the processes of aging and social isolation. According to the RDM, mind–body exercise can work through both external (perceived social support) and internal (psychological resilience) factors to help older adults better cope with life’s challenges, thereby improving their quality of life. Thus, this study aims to investigate the mechanism by which physical activity affects the quality of life of older adults, using perceived social support and psychological resilience as mediating variables, and constructs a chain mediation model of the impact of mind–body exercise on the quality of life of older adults. The research conceptual framework (Figure 1) and research hypotheses are both developed based on the RDM.

## 3 Development of hypotheses

### 3.1 Relationship between mind–body exercise and quality of life

Mind–body exercise focuses on the integration of mind, body, psychology, and behavior. It involves a series of controlled movements and concentration practices to enhance coordination and awareness (26). Due to its significant health benefits, high safety, lack of special equipment requirements, and ease of learning, mind–body exercise has garnered global attention. In China, common forms of mind–body exercise include Tai Chi, Ba duan jin, and Yi jin jing (27), which have significant effects on improving the health status of older adults. Specifically, by enhancing immunity (28), improving sleep quality (29), increasing balance ability (30), and boosting cardiopulmonary function (31), these exercises can reduce the risk of chronic diseases (32), alleviate clinical symptoms such as limb tremors (33), and decrease the incidence of falls and other accidental injuries (34), thereby improving the quality of life and self-care ability of older adults. Furthermore, participation in mind–body exercises is considered a significant investment in mental health. Through breath regulation and meditation, mind–body exercises help older adults to relax and reduce stress. Research indicates that there is a positive correlation between the duration of participation in mind–body exercises and the level of mental health (35), which also aids in enhancing the ability of older adults to adjust to negative emotions. This enables them to more freely cope with various demands of daily life, thereby enjoying a healthier, more vibrant, and satisfying state of living. Thus, this study selects tai chi, ba duan jin, and yi jin jing as mind–body exercises for older adults, all of which can positively influence their quality of life. Based on the preceding analysis, we propose the following hypothesis:

*H1:* Mind–body exercise is positively correlated with the quality of life in older adults.

### 3.2 The mediating of effect perceived social support

Perceived social support plays a crucial effect between mind–body exercise and the quality of life in older adults. Mind–body exercise is a form of physical activity, the essence of which is a type of social interaction, involving not only individuals but also interpersonal communication activities within groups. It is an important way to enhance the level of perceived social support. Research has proven that during the process of participating in physical activities, older adults often gain more longitudinal social support (36). The enhancement of perceived social support can assist older adults in coping with various adverse environments more swiftly. It has a positive buffering effect on individual experiences of anxiety, depression, and distress, preventing the exacerbation of negative emotions and providing continuous regulation for mental health (37). The study results indicate that there is a significant correlation between perceived social support and individual quality of life, and it has a positive predictive effect. The joint action of different types of perceived social support can influence an individual’s physical self-worth, sense of happiness, and motivation (38). A survey conducted by Unsar et al. (39) on older adults aged 60 and above showed that perceived social support is significantly positively correlated with the quality of life and enhancing perceived social support can improve the quality of life in later years. In summary, mind–body exercise is closely related to perceived social support, which further influences the quality of life through perceived social support. As a result, the following hypothesis was advanced:

*H2:* Perceived social support mediates the relationship between mind–body exercise and the quality of life in older adults.

### 3.3 The mediating effect of psychological resilience

Psychological resilience can help individuals successfully cope with stress and improve mental health, playing a crucial role in maintaining the quality of life in older adults (40). According to the theory of psychological defense mechanisms, psychological resilience is associated with mature defense mechanisms. Individuals with higher psychological resilience possess more mature psychological defense abilities, which they can quickly employ at the initial stage of handling challenging or unexpected events, minimizing the impact (41). Studies have shown that compared to younger individuals, older adults, due to their rich life experiences and coping strategies for adversity, may possess equal or even higher levels of psychological resilience (42). High levels of psychological resilience can reduce physical and mental stress in older adults, mitigate the impact of chronic diseases on daily activities, promote physical health, and enhance the quality of life (43, 44). Existing research has demonstrated that the level of physical activity is an important variable affecting an individual’s level of psychological resilience (45). Physical activity contributes to the positive development of psychological resilience, and as physical activity increases, an individual’s psychological resilience significantly strengthens. Higher levels of psychological resilience in older adults are associated with greater autonomy in daily activities, physical activity, and overall physical fitness (46). Previous research has demonstrated that participation in mind–body exercises has a significant positive impact on the psychological resilience of older adults (47), improving their emotional state and mental health levels, thereby enhancing their quality of life. In view of this, we proposed the following hypotheses:

*H3:* Psychological resilience mediates the relationship between mind–body exercise and the quality of life in older adults.

### 3.4 The chain mediating effect of perceived social support and psychological resilience

According to the Rutter Developmental Model (RDM), individuals enhance their quality of life by establishing new cognitive structures, reducing the negative impact of adverse events, and increasing the utilization of internal and external resources. Psychological resilience emphasizes the development and application of individual internal resources, which not only helps older adults maintain a healthy psychological state amidst stress and setbacks, better cope with life’s changes, but also enhances their determination, perseverance, and self-control abilities, promoting the improvement of the quality of life for older adults (48). Perceived social support, as an important external resource, helps to enhance an individual’s ability to cope with trauma, thereby promoting the strengthening of psychological resilience. The more social support older adults perceive, the better they can overcome challenges and adversity with a positive attitude, respond to emergencies with an active mindset, and demonstrate a higher level of psychological resilience (49). Existing research has proven that the perceived social support older adults gain from mind–body exercises is an important factor affecting psychological resilience (50). Perceived social support is a protective factor for psychological resilience, and the process through which psychological resilience functions is the result of the interaction between an individual’s protective factors and high-risk situations (51). The more perceived social support older adults obtain through participation in mind–body exercises, the stronger their psychological resilience becomes, and consequently, their quality of life is enhanced. Accordingly, the research hypothesis was formulated:

*H4:* Perceived social support and psychological resilience have a chain mediating effect between mind–body exercise and the quality of life in older adults.

## 4 Materials and methods

### 4.1 Research design

In order to obtain the raw data required for the study, researchers designed a self-administered questionnaire based on existing literature and validated data collection tools, focusing on assessing older adults’ participation in mind–body exercises, perceived social support, psychological resilience, and quality of life. Since the participants are from the Chinese population, the questionnaire is conducted in Chinese. The tools used in this study are scales that have been scientifically translated and validated, suitable for Chinese older adults, with reliable validity and reliability. Before the final survey, the research team conducted a preliminary assessment (pilot test) with 20 participants to ensure the feasibility of the questionnaire and to test the response rate. Through the pilot study, researchers made adjustments and edits to some of the questions in the questionnaire to maximize the accuracy and comprehensiveness of the responses. From March 25 to May 3, 2024, the research team conducted a questionnaire survey among older adults participating in mind–body exercises (Tai Chi, Ba Duan Jin, Yi Jin Jing) in 13 districts of Beijing, China. The questionnaire included: the Physical Activity Rating Scale (PARS-3), the WHOQOL-BREF scale, the Perceived Social Support Scale (PSSS), and the Chinese version of the Connor-Davidson Resilience Scale (CD-RISC). All data collection was ensured to be conducted under uniform conditions to minimize data bias, with each questionnaire taking approximately 10–20 min to complete. All invitees participated voluntarily, and the project was approved by the Ethics Committee of Beijing Sport University (2024145H).

### 4.2 Inclusion criteria

Inclusion criteria for participants in this study (1): older adults aged 60 and above (2); older adults with good reading and comprehension abilities who can communicate normally (3); older adults with experience in practicing mind–body exercises (tai chi, ba duan jin, yi jin jing) (4); older adults willing to participate in the survey after fully understanding its purpose. Exclusion criteria (1): Individuals with severe hearing or speech impairments (2); Individuals with abnormal mental states (3); Individuals with severe physical diseases, extreme frailty, or disabilities that prevent them from cooperating with the survey. A total of 1,239 offline questionnaires were collected in this study, and after applying the exclusion and inclusion criteria, 1,087 valid questionnaires were obtained, resulting in an effective recovery rate of 87.73%.

### 4.3 Measurement tool design and reliability testing

#### 4.3.1 Mind–body exercises level

The level of mind–body exercises was measured using the Physical Activity Rating Scale (PARS-3) developed by Liang et al. (52). The PARS-3 includes three items: exercise intensity, exercise duration, and exercise frequency, each scored using a 5-point Likert scale ranging from 1 to 5. The total score of the scale is calculated using the formula: Frequency score × (Duration score - 1) × Intensity score, with a range of 0 to 100. Higher scores indicate greater exercise intensity, higher frequency, and longer duration. The criteria for exercise volume levels are as follows: 19 points or below indicates mild exercise volume; 20–42 points indicate moderate exercise volume; 43 points or above indicate intense exercise volume. In this study, the Cronbach’s *α* coefficient for the scale was 0.850.

#### 4.3.2 Quality of life scale

The Quality of Life Scale used in this study is the WHOQOL-BREF scale developed by the World Health Organization based on its definition of quality of life (53). This scale consists of 26 items that can be categorized into four domains: physical health (8 items), psychological health (6 items), social relationships (3 items), and environment (9 items). Each item is scored on a Likert 5-point scale, ranging from “never” to “always,” with higher scores indicating a higher level of quality of life. The Cronbach’s *α* coefficient for this study is 0.970.

#### 4.3.3 Perceived social support scale

The Perceived Social Support Scale (PSSS) was developed by Zimet et al. in 1988 (54). This scale consists of 12 items, encompassing three dimensions: family support (4 items), friend support (4 items), and other support (4 items). It measures the degree of perceived support from family, friends, and others, with the total score reflecting the overall level of perceived social support. The items are scored on a 7-point Likert scale, ranging from “strongly disagree” to “strongly agree.” Total scores between 12 and 36 indicate low support; scores between 37 and 60 indicate moderate support; scores between 61 and 84 indicate high support. Higher total scores indicate higher levels of perceived social support. The Cronbach’s *α* coefficient for this study is 0.950.

#### 4.3.4 Psychological resilience scale

The Psychological Resilience Scale used in this study is the Chinese version of the Connor-Davidson Resilience Scale (CD-RISC), translated by Yu and Zhang (55). This scale consists of 25 items, covering three dimensions: tenacity (13 items), strength (8 items), and optimism (4 items). Each item is scored on a Likert 5-point scale, ranging from “never” to “always,” with higher scores indicating higher levels of psychological resilience. The Cronbach’s *α* coefficient for this study is 0.950.

#### 4.3.5 The covariates of the study

In this study, the independent variable is mind–body exercise, the dependent variable is the quality of life in older adults, and the mediator variables are perceived social support and psychological resilience. Research findings on quality of life have indicated that the gender and age of the subjects have become variables that scholars need to control in their studies (56). In addition, quality of life is significantly associated with education level (57), income status (58), and baseline health conditions (59). Therefore, these variables were used as control variables in the study, adjusted during data analysis to minimize their impact on the main results, ensuring that the research focus is on the effects of mind–body exercise on older adults’ perceived social support, psychological resilience, and quality of life. This study refers to Sun Wen’s age classification (60), Zhang Wen’s education classification (61), and the classification of monthly income in the China Longitudinal Aging Social Survey (62).

#### 4.3.6 Statistical analysis

After organizing the valid questionnaire data, the data was analyzed using SPSS 26.0 software. Correlation analysis, linear regression analysis, and other methods were used to examine the impact of mind–body exercise, perceived social support, and psychological resilience on the quality of life in older adults. The Amos 24.0 software package was used to validate the model and test the construct validity of the scales. Currently, the Bootstrap method is commonly used to test mediation effects. This method involves resampling from the original sample and using a 95% confidence interval to test the significance of the mediation effect coefficients. Therefore, this study used the Bootstrap method to test whether perceived social support and psychological resilience mediate the relationship between mind–body exercise and the quality of life in older adults, as well as whether there is a chain mediation effect of perceived social support and psychological resilience in the relationship between mind–body exercise and the quality of life in older adults.

## 5 Results

### 5.1 Reliability and validity testing

The scales used in this study were adapted from previous research questionnaires, so it is necessary to verify their reliability and validity. To further test the convergent validity and reliability of the scales, Average Variance Extracted (AVE) and Construct Reliability (CR) were used as evaluation parameters. As shown in Table 1, the AVE values for all factors are greater than 0.5, indicating good model convergence; the CR values for all factors are greater than 0.7, indicating that the items in each scale consistently explain the latent variables, demonstrating good construct reliability. In summary, the questionnaire used in this study has high reliability and validity.

**Table 1:** Validity and reliability test of the questionnaires.

Variable	CR	AVE
QOL	0.931	0.771
MBE	0.854	0.662
PSS	0.842	0.643
PR	0.807	0.585

CR, composite reliability; AVE, average variance extracted; QOL, quality of life scale; MBE, mind–body exercise; PSS, perceived social support; PR, psychological resilience.

### 5.2 Common method bias testing

To avoid common method bias, an anonymous coded evaluation method was used during the testing process to control the sources of common method bias from the procedure. Additionally, SPSS 26.0 was used to conduct an exploratory factor analysis on all test items using Harman’s single-factor test. The results showed that there were nine factors with eigenvalues greater than 1, and the variance explained by the first factor was 36.44%, which is below the critical standard of 40%, indicating that there is no serious common method bias in this study.

### 5.3 Descriptive statistics and correlation analysis

Table 2 indicates that the study participants numbered 1,087, with a majority consisting of older adults with chronic diseases, totaling 694 individuals (63.8%), while those without chronic diseases accounted for 393 (36.1%). The gender distribution was balanced, with 548 males (50.4%) and 539 females (49.6%). The age distribution varied across different age groups, notably with 60–64 years old participants making up 35%, followed by 65–69 years old (34.1%), 70–74 years old (22%), 75–79 years old (4.1%), and those aged 80 and above representing 4.8%. The educational background of the participants was diverse, with the highest level of education being undergraduate and above (32.29%), followed by college and higher vocational education (23.82%), high school or technical secondary school (18.67%), middle school (17.01%), and elementary school and below (8.18%). In terms of income levels, the highest percentage was for those earning above 5,001 (40.66%), followed by 3,501–5,000 (35.97%), 1,501–3,500 (18.67%), and below 1,500 (4.69%).

**Table 2:** Participant demographics.

Demographic category	Frequency	Percent%
Gender		
Male	548	50.4
Female	539	49.6
Age		
60–64	380	35
65–69	371	34.1
70–74	239	22
75–79	45	4.1
80 years and above	52	4.8
Education level		
Elementary school and below	89	8.18
Middle school	185	17.01
High school or technical secondary school	203	18.67
Junior college or higher vocational education	259	23.82
Undergraduate and above	351	32.29
Monthly income		
Below 1,500	51	4.69
1,501–3,500	203	18.67
3,501–5,000	391	35.97
Above 5,001	442	40.66

This study focuses on examining the overall scores of the model indicators, without delving into the sub-dimensions of each indicator. Pearson correlation analysis was conducted using the average scores of each variable. The descriptive statistics and correlation analysis results for the four variables: mind–body exercise, perceived social support, psychological resilience, and the quality of life in older adults, are shown in Table 3. The results indicate significant positive correlations between each pair of the four variables. Additionally, the correlation analysis revealed that mind–body exercise and quality of life in older adults are significantly correlated (*r* = 0.549, *p* < 0.01); mind–body exercise and perceived social support are significantly positively correlated (*r* = 0.402, *p* < 0.01); mind–body exercise and psychological resilience are significantly correlated (*r* = 0.393, *p* < 0.01); perceived social support and quality of life in older adults are significantly correlated (*r* = 0.474, *p* < 0.01); psychological resilience and quality of life in older adults are significantly correlated (*r* = 0.527, *p* < 0.01); perceived social support and psychological resilience are significantly correlated (*r* = 0.281, *p* < 0.01). In summary, the significant correlations among the variables provide preliminary evidence for the hypotheses proposed in this study.

**Table 3:** Descriptive statistics and correlations for primary variables.

Variable	*M*	S.D.	MBE	PSS	PR	QOL
MBE	9.80	2.81	1			
PSS	53.69	13.88	0.402**	1		
PR	82.99	18.14	0.393**	0.281**	1	
QOL	83.73	21.92	0.549**	0.474**	0.527**	1

*n* = 1,078; M, mean; S.D., standard deviation. *p < 0.05; **p < 0.01; QOL, quality of life scale; MBE, mind–body exercise; PSS, perceived social support; PR, psychological resilience.

This study employed univariate analysis of variance (ANOVA) to examine the differential impact of varying levels of physical activity on the quality of life among older adults. As can be seen from Table 4, there is a significant difference in quality of life across samples with different amounts of physical activity (*p* < 0.05), indicating a variability in the quality of life among these samples. The level of physical activity shows a significant effect on quality of life at the 0.01 level (*F* = 148.400, *p* = 0.000). Comparative differences reveal that participation in moderate to Intense exercise is more conducive to enhancing the quality of life for older adults.

**Table 4:** Anova results of the effects of physical exercise mind–body exercise on the quality of life in older adults.

Variable	Exercise amount (M + S.D.)			*F*	*p*
	Intense exercise (*n* = 195)	Moderate exercise (*n* = 483)	Mild exercise (*n* = 409)		
The quality of life	88.733 ± 18.499	94.494 ± 21.708	72.590 ± 17.709	148.4	0.000**

*p < 0.05; **p < 0.01.

### 5.4 Analysis of mediation effects

To verify the chain mediating effect of perceived social support and psychological resilience in the relationship between mind–body exercise and the quality of life in older adults, AMOS 24.0 software was used to perform a fit analysis of the conceptual framework chain mediation model. Following the mediation effect testing process proposed by Marsh et al. (63), Table 5 shows the standard results of the fit indices: χ2/df < 5, GFI > 0.9, RMSEA <0.10, CFI > 0.9, NFI > 0.9, TLI > 0.9. The model parameters meet the fit requirements, indicating that the mediation model of mind–body exercise and the quality of life in older adults is reasonable.

**Table 5:** Questionnaire model fitting indicators.

	*X* ^2^	df	*χ*^2^/*df*	RMSEA	CFI	GFI	NFI	TLI	IFI
Model	234.304	59	3.971	0.052	0.968	0.968	0.973	0.973	0.98

RMSEA, root mean square error of approximation; CFI, comparative fit index; GFI, goodness-of-fit index; NFI, normed fit index; TLI, Tucker-Lewis index. IFI, increased fit index.

**Figure 2:**
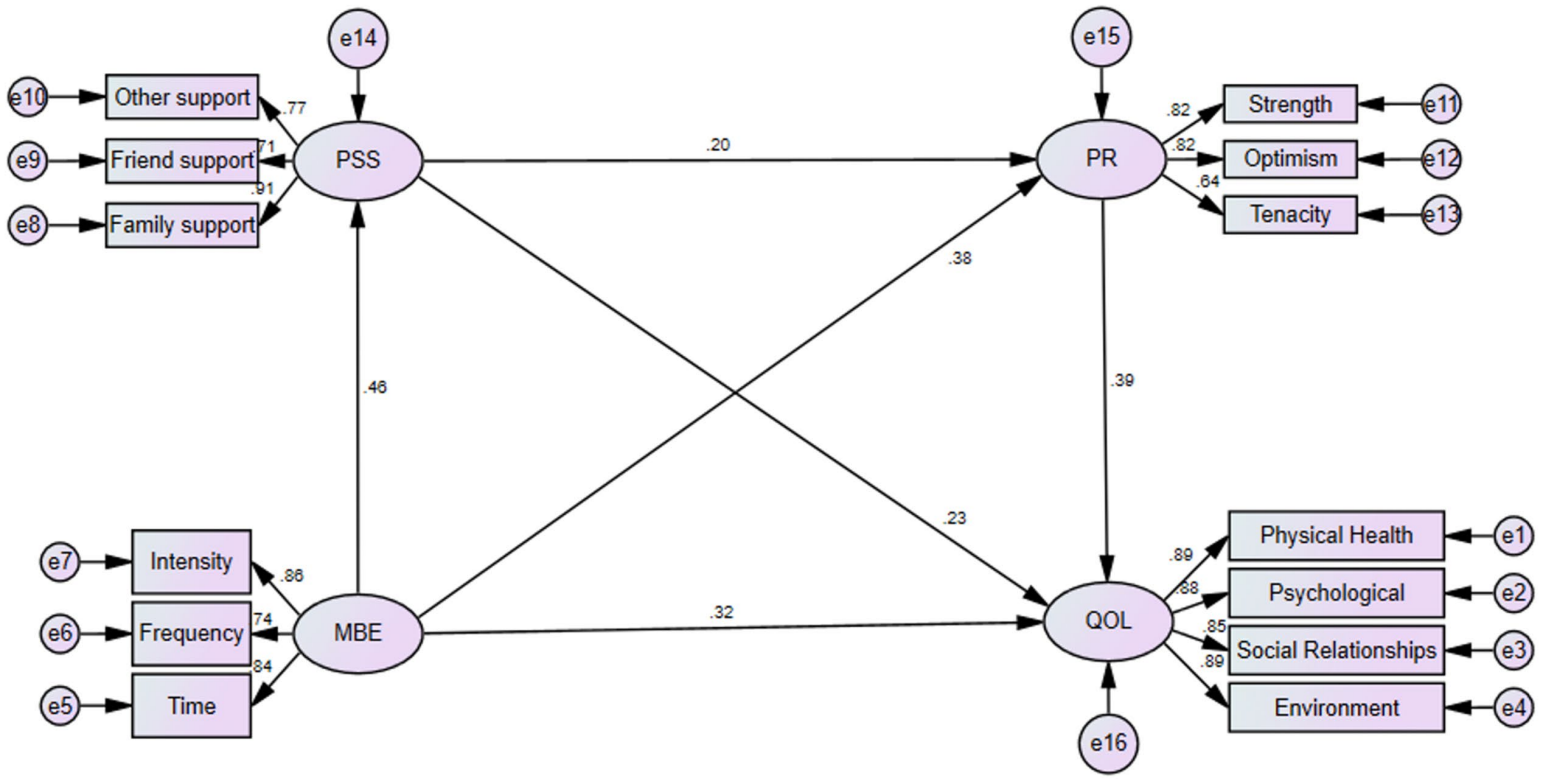
Intermediary model diagram. QOL, quality of life scale; MBE, mind–body exercise; PSS, perceived social support; PR, psychological resilience.

Furthermore, the standardized path coefficient model of the impact of mind–body exercise on the quality of life in older adults is shown in Figure 2, which indicates that the standardized path coefficient for mind–body exercise → quality of life in older adults is significant (*β* = 0.32, *p* < 0.001), indicating a significant positive effect of mind–body exercise on the quality of life in older adults, supporting hypothesis 1. The path coefficients for mind–body exercise → perceived social support (*β* = 0.46, *p* < 0.001) → quality of life in older adults (*β* = 0.23, *p* < 0.001) are significant, indicating that perceived social support mediates the relationship between mind–body exercise and the quality of life in older adults, supporting hypothesis 2. The path coefficients for mind–body exercise → psychological resilience (*β* = 0.38, *p* < 0.001) → quality of life in older adults (*β* = 0.39, *p* < 0.001) are significant, indicating that psychological resilience mediates the relationship between mind–body exercise and the quality of life in older adults, supporting hypothesis 3. The path coefficients for mind–body exercise → perceived social support (*β* = 0.46, *p* < 0.001) → psychological resilience (*β* = 0.20, *p* < 0.001) → quality of life in older adults (*β* = 0.39, *p* < 0.001) are significant, indicating that perceived social support and psychological resilience have a chain mediating effect between mind–body exercise and the quality of life in older adults, supporting hypothesis 4.

The bias-corrected non-parametric percentile Bootstrap method was used to evaluate the significance of individual mediation effects to confirm the mediating effect of perceived social support and psychological resilience. Hayes recommends that the number of resamples for Bootstrap mediation effect testing should be at least 1,000 for the original sample (64). Bootstrap mediation effect test results show that if the Bootstrap test CI does not include 0, the indirect effect is considered established (65). In this study, 5,000 Bootstrap samples were used to test the mediation effect, determining a 95% confidence interval (CI). As shown in Table 6, the direct effect of mind–body exercise on the quality of life in older adults is significant (direct effect = 2.173, 95% CI [1.697, 2.703]). The indirect effects include three significant mediation pathways: mind–body exercise → perceived social support → quality of life (indirect effect = 0.733, 95% CI [0.500, 0.980]); mind–body exercise → psychological resilience → quality of life (indirect effect = 1.062, 95% CI [0.832, 1.299]); mind–body exercise → perceived social support → psychological resilience → quality of life (indirect effect = 0.245, 95% CI [0.141, 0.375]). In summary, the 95% confidence intervals for the three mediation paths do not include 0, indicating that the path coefficients in this model are significant. This demonstrates that perceived social support and psychological resilience mediate the relationship between mind–body exercise and the quality of life in older adults, and that perceived social support and psychological resilience play a chain mediation effect.

**Table 6:** Test results of mediation effects.

Effect	Parameter	Estimate	BootSE	Bootstrap LLCI	Bootstrap ULCI
Direct effect	MBE → QOL	2.173	0.254	1.697	2.703
Indirect effect	MBE → PSS → QOL	0.733	0.125	0.5	0.98
	MBE → PR → QOL	1.062	0.166	0.832	1.299
	MBE → PSS → PR → QOL	0.245	0.061	0.141	0.375
Total effect	MBE → PSS → PR → QOL	4.212	0.241	3.728	4.67

The Bootstrap sample size is set at 5000.

In order to explore the mediating effect of perceived social support from various sources on the relationship between mind–body exercise and the quality of life in older adults, family support, friend support, and other support were used as independent variables, while quality of life was considered as the dependent variable for linear regression analysis. Table 7 indicates that the model equation is: Quality of Life = 44.387 + 1.130*Family Support +0.611*Friend Support +0.466*Other Support, with an R-squared value of 0.228, suggesting that family support, friend support, and other support can influence the enhancement of quality of life. The model passes the *F-test* (*F* = 106.821, *p* < 0.05), indicating that at least one of the factors—family support, friend support, or other support—has a significant impact on the quality of life. Additionally, the examination of multicollinearity in the model revealed that all VIF values were less than 5, indicating the absence of multicollinearity issues. Furthermore, the Durbin-Watson (D-W) value was around 2, suggesting that there is no autocorrelation in the model, and the sample data do not exhibit any interrelationships, indicating a good model fit. A detailed analysis showed that the regression coefficient for family support was 1.130 (*t* = 6.676, *p* < 0.01), which means that family support has a significant positive impact on the quality of life. Friend support has a regression coefficient value of 0.611 (*t* = 4.128, *p* < 0.01), indicating that friend support significantly and positively influences the quality of life. Other support has a regression coefficient value of 0.466 (*t* = 2.940, *p* < 0.01), indicating that other support also significantly and positively affects the quality of life. In summary, both family support, friend support, and other support significantly and positively impact the quality of life. The magnitude of the standardized coefficients suggests a ranking of their relative influence as family support > friend support > other support.

**Table 7:** Linear regression analysis results (*n* = 1,087).

	Unstandardized coefficients		Standardized coefficients	*t*	*p*	Collinearity diagnostics	
	*B*	Standard error	*Beta*			VIF	Tolerance
Constant	44.387	2.37	–	18.73	0.000**	–	–
Family support	1.13	0.169	0.281	6.676	0.000**	2.494	0.401
Friend support	0.611	0.148	0.146	4.128	0.000**	1.752	0.571
Other support	0.466	0.159	0.112	2.94	0.003**	2.047	0.489
*R* ^2^	0.228						
Adjusted *R*^2^	0.226						
*F*	*F* (3,1,083) =106.821, *p* = 0.000						
D-W值	2.071						

*p < 0.05; **p < 0.01.

## 6 Discussion

This study explored the impact of mind–body exercise on the quality of life in older adults, with a particular focus on the serial mediating roles of perceived social support and psychological resilience. The findings confirm a significant positive correlation between mind–body exercise and the quality of life in older adults. This is consistent with previous research findings (66, 67). Mind–body exercise focuses on enhancing physical strength, flexibility, and balance, thereby avoiding potential injuries associated with high-intensity exercise. Through movement, breathing, and meditation, it alleviates psychological stress and achieves overall coordination and balance of the body and mind, demonstrating significant effectiveness in improving both physical and mental health (68). Consequently, it can effectively enhance the quality of life in older adults. The study further found that participation in moderate to high-intensity mind–body exercise is more conducive to enhancing the quality of life for older adults. This improvement is not only reflected in the enhancement of physical function but also manifested in various aspects of the daily lives of older adults. Engaging in such exercise can foster the habit of maintaining a long-term exercise routine, thereby continuously preserving a state of good health, boosting the confidence of older adults, and promoting emotional stability. Consequently, this leads to an overall increase in life satisfaction and well-being, resulting in a significant enhancement in the quality of life for older adults.

Mind–body exercise not only directly affects the quality of life in older adults but also indirectly influences it by positively impacting perceived social support. Mind–body exercise has a beneficial effect on the level of perceived social support, and the amount of exercise is significantly positively correlated with perceived social support (69). Regular participation in mind–body exercise by older adults not only helps them communicate with others, establish interpersonal relationships, and emotional connections during the exercise process, but also promotes or improves the structure of their social networks (70). Through these social interactions, older adults can enhance their sense of social connection, gain more support from family, friends, and other social sources, which helps alleviate their feelings of loneliness and increase their sense of social belonging. This, in turn, further improves their quality of life, consistent with previous research findings (71). Additionally, this study also found that perceived social support from different sources can have varying impacts on the quality of life in older adults, with family support having the greatest influence on quality of life. This may be related to the concept of filial piety in Chinese culture, where the family (especially spouses and children) plays a significant role in providing emotional support and practical assistance to older adults, particularly in alleviating negative emotions.

At the same time, mind–body exercise can also indirectly affect the quality of life in older adults by having a positive impact on psychological resilience. Mind–body exercise is a protective factor that promotes the development of individual psychological resilience and has a significant predictive role in psychological resilience (72). During the participation in mind–body activities, older adults may fulfil their basic needs for self-competence and intimate relationships, and the satisfaction of these basic needs can promote the natural development of efficacy and autonomy in individuals. This helps to alleviate perceived stress and emotional suppression, leading to better psychological performance in coping with adversity and challenges (73). As the level of psychological resilience increases, the quality of life for older adults also increases. Psychological resilience has a significant positive predictive effect on the quality of life, which is consistent with existing research findings (74). Individuals with high psychological resilience often maintain a more positive attitude when facing life pressures. Relevant surveys indicate that older adults with high psychological resilience continue to enhance their perceived value through various social activities even after retirement, pursuing higher goals, which in turn improves their quality of life (75).

There is a relatively stable systemic association between perceived social support and psychological resilience in older adults (76). The more social support individuals perceive, the more they experience respect, support, and understanding emotionally, the higher their level of inner satisfaction, and the stronger their ability to resist adversity, resulting in a higher level of psychological resilience (77). Therefore, individuals with high perceived social support tend to have higher levels of psychological resilience. Older adults with higher levels of psychological resilience have greater coping abilities in life, thereby achieving a higher quality of life, which is consistent with previous research findings (78). The enhancing effect of mind–body exercise on the quality of life for older adults is multidimensional, as it promotes the well-being of older adults from both external and internal perspectives. From the external environmental factors, older adults can gain positive social support, including mental and material assistance, during their participation in mind–body exercise. This not only meets their living needs but also helps in developing certain psychological resilience traits as internal factors (79). These internal and external factors interact with each other to comprehensively promote the improvement of the quality of life for older adults. The results of this study further demonstrate that mind–body exercise can collectively influence the quality of life in older adults through the serial mediating effects of perceived social support and psychological resilience.

## 7 Conclusion

This study reveals the impact of mind–body exercise on the quality of life in older adults and provides an in-depth analysis through the serial mediating effects of perceived social support and psychological resilience. The results indicate that mind–body exercise has a positive influence on the quality of life for older adults, with those who regularly participate in such activities not only demonstrating higher levels of quality of life but also showing significant improvements in perceived social support and psychological resilience. The study found that perceived social support, as an external protective resource, has a significant mediating effect between mind–body exercise and quality of life in older adults, with family support playing a particularly important role in promoting the quality of life in older adults. Additionally, psychological resilience, as an internal protective resource, serves as an important mediating variable. When facing challenges such as aging, illness, and social isolation, it helps older adults maintain a positive attitude toward life through active emotional regulation and coping strategies, thereby promoting the improvement of quality of life. This study is the first to validate the chain mediating effect of perceived social support and psychological resilience between mind–body exercise and the quality of life in older adults. Mind–body exercise not only improves the quality of life for older adults but also enhances their life satisfaction and sense of well-being through the elevation of perceived social support and psychological resilience. By effectively utilizing perceived social support and strengthening psychological resilience, older adults can better cope with life’s challenges, maintain a positive attitude toward life, and thereby achieve a comprehensive improvement in the quality of life. The chain mediating model proposed in this study integrates the relationship between perceived social support, psychological resilience, and the quality of life in older adults, providing a new perspective for us to more comprehensively understand how mind–body exercise affects the quality of life in older adults through internal mechanisms. This finding not only enriches the existing literature but also provides a theoretical basis for the application of mind–body exercise in the field of health promotion for older adults.

## 8 Implications and limitations

### 8.1 Implications

This study explores the mechanism by which mind–body exercise impacts the quality of life in older adults, introducing perceived social support and psychological resilience as mediating variables, thus expanding the existing research on the impact of mind–body exercise on individual quality of life. Mind–body exercise plays a crucial role in improving physical and mental health, effectively helping older adults gain necessary social connections and emotional support, while also reducing negative emotions such as loneliness, anxiety, and depression. By accumulating more social capital, it improves overall quality of life. The study of the chain mediating effect of perceived social support and psychological resilience provides new perspectives and practical approaches for enhancing the quality of life in older adults. Firstly, when aiming to improve the quality of life in older adults, it is essential to adopt more comprehensive intervention measures, taking into account the roles of mind–body exercise, perceived social support, and psychological resilience, to create a positive living environment and healthy lifestyle for older adults (80). Secondly, the community environment should meet the psychological and social needs of older adults, enhancing their social support network with systems composed of family members, friends, and community volunteers. Educating older adults on how to effectively perceive and utilize social support can enhance their positive emotional experiences and further improve their psychological health and life satisfaction. Lastly, attention should be paid to the mechanisms of psychological resilience formation in older adults, with regular, broad-ranging health education and psychological counseling to promote more comprehensive psychological and social growth. This will increase positive emotions and encourage them to have greater confidence in their daily lives.

### 8.2 Limitations

Despite providing strong evidence of the positive impact of mind–body exercise, perceived social support, and psychological resilience on improving the quality of life in older adults, this study has several limitations. First, the study only explored three types of mind–body exercises: tai chi, ba duan jin, and yi jin jing, while neglecting others such as yoga, Pilates, and meditation. The different types may have varying effects on older adults, this study did not fully consider these differences, suggesting that future research should refine the study design to control for the variables of different exercise types. Second, this study employed a cross-sectional design to explore the mechanism of how mind–body exercise affects the quality of life in older adults, which can only reveal correlations and not causations. Future research could use longitudinal tracking designs to further test and confirm the findings of this study. Finally, the research sample was limited to a specific area in Beijing, China, which may not fully represent all older adults. Differences in understanding and manifestations of these variables across regions could affect the generalizability of the results. Future research should consider expanding the sample collection area to improve the study’s conclusions.

## Data Availability

The original contributions presented in the study are included in the article/supplementary material, further inquiries can be directed to the corresponding author/s.
